# Predictors of Overweight/Obesity Among Preschool Children in Arba Minch City, Southern Ethiopia: Does Consumption of Sweet Food Predispose to Overweight/Obesity?

**DOI:** 10.3389/ijph.2024.1607017

**Published:** 2024-10-01

**Authors:** Simegn Wagaye Kefene, Tamirat Gezahegn Guyo, Darik Temesgen Assefa, Yilma Chisha, Muluken Bekele Sorrie

**Affiliations:** ^1^ Department of Public Health, Arba Minch College of Health Sciences, Arba Minch, Ethiopia; ^2^ School of Public Health, College of Medicine and Health Sciences, Arba Minch University, Arba Minch, Ethiopia

**Keywords:** overweight, obesity, preschool children, Arba Minch, South Ethiopia

## Abstract

**Objectives:**

Childhood overweight/obesity is a serious public health problem in the world today specially low-and-middle income countries like Ethiopia. This study aimed to assess the prevalence of overweight/obesity and associated factors among preschool children.

**Methods:**

A community-based cross-sectional study was conducted among preschool children aged 3–5 years in Arba Minch city from April to June 2022. A multistage sampling method was used to select 577 study participants. Data were collected using a structured and pretested questionnaire. A binary logistic regression model was used for analysis. An adjusted odds ratio (AOR) with a 95% confidence interval (CI) was used to assess the strength of the association.

**Results:**

The combined prevalence of overweight/obesity was 23.9% (95% CI: 20.5, 27.6). Age of 36–47 months, family size < five, watching TV/video for ≥2 h, and consuming sweet foods, were factors associated with being overweight/obese among preschool children.

**Conclusion:**

Overweight/obesity was predicted by consuming sweet foods, long time TV/video watching, and large family size in the study area. Special attention should be given to child feeding practices and reducing sedentary lifestyles.

## Introduction

Obesity and overweight are characterized by abnormal or excessive fat buildup that may have negative health effects [1]. Body mass index (BMI) is a simple index of weight-for-height that is commonly used to classify overweight and obesity, and childhood obesity and overweight were defined as body mass index (BMI) for Age Z-scores between +2 SD and +3 SD and above +3 SD, respectively [1]. This form of malnutrition (overweight or obesity) comes from energy intake from meals and drinks that exceed children’s energy requirements [2]. Previously, overweight/obesity was considered an issue for high-income nations (developed countries), but recently, it has become a concern for low- and middle-income countries particularly urban settings [3].

Globally, in 2019, about 38.2 million children under the age of 5 were overweight or obese [1]. In Africa, the number of overweight children under the age of 5 has increased by nearly 24% since 2000 [1]. Evidence from multilevel analysis from sub-Saharan Africa (SSA) revealed that the prevalence of overweight/obesity among under-five-year-old children was 5.10%, with the highest prevalence in the southern and eastern parts of the continent [4]. Ethiopia is one of the SSA countries located in the eastern part, and this critical public health problem is highly increasing and ranges from 2.14% to 16.6% among children [5–8].

Obesity is the predominant risk factor for the world’s main causes of morbidity, and mortality, and children affected by this problem have an increased risk of developing non-communicable diseases like cardiovascular disease, metabolic disorders, and orthopedic disorders as compared to children of normal weight [9]. The economic activities of families and countries as a whole are impacted by childhood overweight/obesity, which results in direct and indirect healthcare expenses [10]. Additionally, a child’s poorer quality of life and poor academic achievement are associated with childhood obesity [11]. Obesity is also responsible for the rising in disability-adjusted life years (DALYs) in middle-income-and-low- countries [12]. Children who are overweight or obese have a greater risk of remaining obese as adults, and losing the excess weight is more difficult for these adults once they become overweight or obese [13]. Childhood obesity is linked to a higher chance of developing non-communicable diseases (NCDs) earlier in life, which may last throughout adulthood, and this in turn leads to early mortality and disability in adulthood [13]. In addition, the management of obesity in adults is challenging and even ineffective, particularly in the absence of a known organic etiopathogenesis [14]. Therefore, prevention of childhood overweight and obesity can be more worthwhile, providing better chances for minimizing its lifelong effects and complications.

Evidence from previously conducted studies showed that factors associated with overweight or obesity include the wealth index status of the family, birth weight, the behavioral and physical activity status of the child, such as watching TV and playing electronic games, and a high dietary diversity score [6, 15–18].

Even if different intervention measures are taken in different parts of the world, including early identification of cases by screening, behavioral change, and dietary modification implemented among children, the occurrence of this public health problem is increasing [19, 20]. Moreover, there is a variation in the overweight/obesity prevalence in Ethiopia based on the different sociodemographic and economic characteristics across the different regions of the nation, which might affect the decision-making process during intervention strategies designing by policymakers and this enhances the need for estimating the prevalence in different settings. There is no evidence in the study area regarding the prevalence of overweight and obesity in this age group. Therefore, this study was aimed at assessing the prevalence of overweight/obesity and its associated factors among preschool children in Arba Minch City, South Ethiopia.

## Methods

### Study Design, Period, and Settings

A community-based cross-sectional study was conducted among preschool children in Arba Minch city. The study was undertaken from April 30 to June 05, 2022. Arba Minch city is located 505 km away from Addis Ababa, the capital city of Ethiopia, and 275 km away from Hawassa, the capital city of the South Nations, Nationalities, and People’s Region. Administratively, the city has been divided into seven Kebeles (small administrative units or neighborhoods), and each Kebele encompassing three Sub-Kebeles. The total population of the city is estimated around 210,811. There are 32,908 under five children in the city. Of which 15,389 were aged 3–5 years. The city has four public health facilities (one general hospital, one primary hospital, and two health centers) [21].

### Population

All preschool children with their mothers or caregivers who live in Arba Minch city were the source population. All randomly selected preschool children with their mothers or care givers who live in the selected Kebeles of Arba Minch city were the study population. All preschool children with their mothers or caregivers who were living in the selected Kebeles for about 6 months or more and available during the data collection period were included in the study. Preschool children who were with known chronic diseases (having heart, liver, and kidney diseases) and with edema during the data collection period were excluded from the study.

### Sample Size and Sampling Procedure

The sample size was determined using OpenEpi by considering 95% confidence level, 5% margin of error, 80% power, 1:1 ratio of unexposed to exposed, and the 17.5% prevalence of the outcome (overweight/obesity) among exposed and 5.7% among unexposed by taking sweet food consumption as an exposure variable from a study done in Gondar City, Northwest Ethiopia [6]. After multiplying the calculated sample size with a design effect of 2 and adding a 10% non-response rate, the final sample size was found to be 577.

**FIGURE 1:**
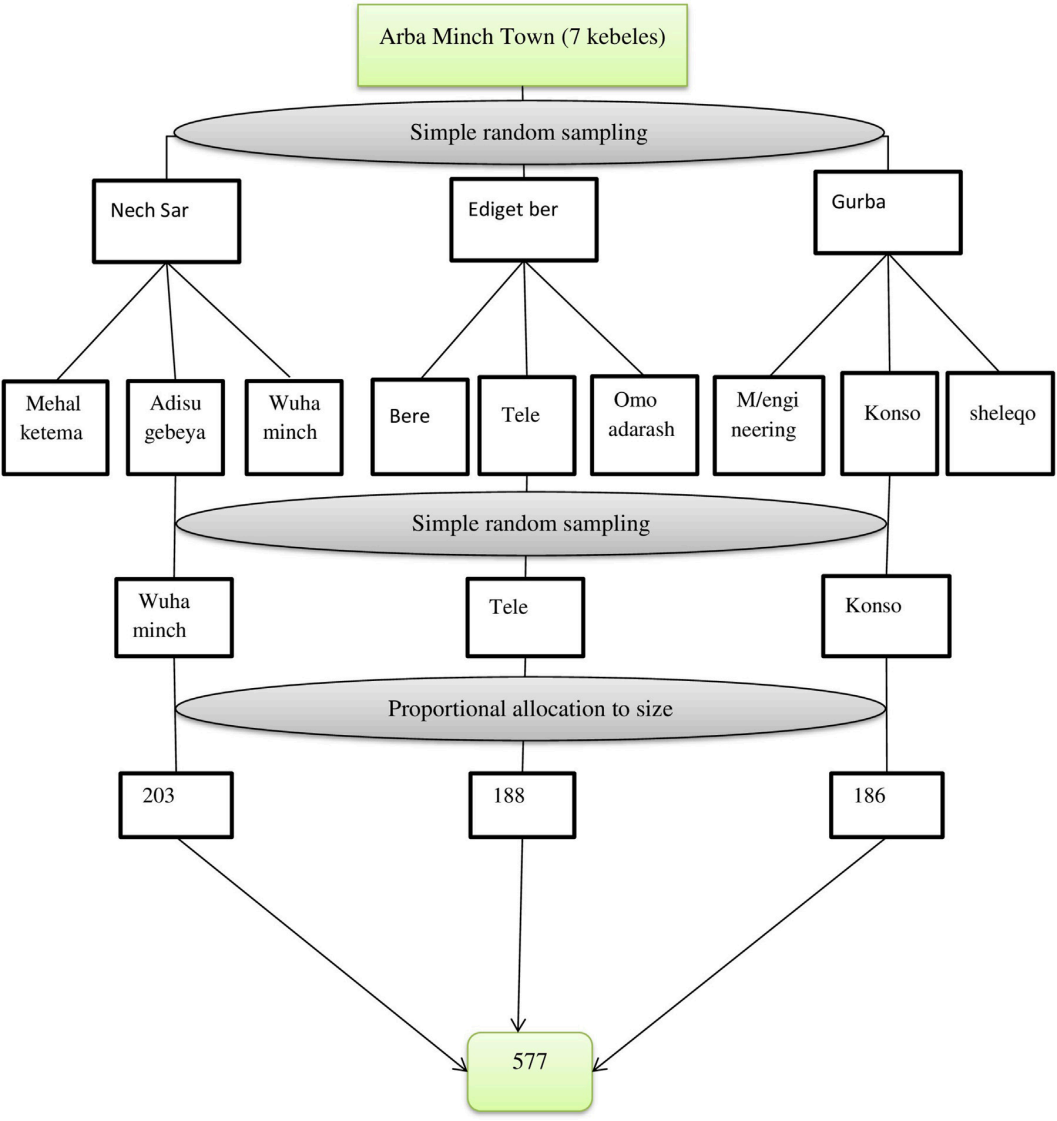
Schematic presentation of the sampling procedure to assess obesity/overweight among preschool children in Arba Minch City, South Ethiopia, 2022 (Predictors of Overweight/Obesity among Preschool Children in Arba Minch City, Southern Ethiopia, 2022: Does Consumption of Sweet Food Predispose to Overweight/Obesity?).

A Multistage sampling technique was employed to recruit a total of 577 study participants during the study period. There were seven Kebeles in the city. From those Kebeles, three (Wuha Minch, Tele, and Ediget Ber) were selected randomly by using the lottery method. Then, from the selected Kebeles, three sub-Kebeles were selected by lottery method. Samples were proportionally allocated to those sub-Kebeles according to their size. To find eligible participants, systematic random sampling methods were applied (Figure 1). Initially, the family folder of every Kebele was checked for listings of households. It was determined that the Kth value was four. Next, every four households were considered, and a number between one and four was chosen to begin the data collection.

### Data Collection Methods and Instruments

Data was collected by using an interviewer-administered, structured, and pretested questionnaire. The questionnaire contains items on sociodemographic and economic characteristics of the child and parents, behavioral characteristics of the child, feeding practices of the child, and dietary diversity score of the child. Parents or caregivers’ knowledge about the consumption of healthier food was asked by using 6 questions and scored based on the mean value. Child dietary diversity scores were determined by employing the multiple pass 24-h recall method. First, parents or caregivers were asked to list foods consumed during the previous 24 h, and then the interviewers were probed for foods possibly forgotten. They were asked the time and occasion of each consumed food. Finally, the interviewer requested them to remember if anything else was consumed. Then the listed food items were categorized into seven food groups which were adopted from WHO 2010 [22]. Wealth indices of the households were measured by asking questions about their household assets. The Global Physical Activity Questionnaire was adapted from WHO to assess the physical activity level of children [23].

Weight and height of the preschool children were measured by using calibrated and standardized equipment. Height was measured with a stadiometer seca (Model 869, Seca) and recorded to the nearest 0.1 cm by putting the children in Frankfurt position and removing their shoes and hair braids. Weights were measured by using seca digital weighing and the scales were calibrated regularly by portable standard weight of 1 kg daily and adjusted to zero after every measurement and recorded to the nearest 0.1 kg by removing the heavy cloths. The questionnaires were uploaded to an open data kit. Six data collectors, and those who had a diploma in nursing, participated in the data collection process and one public health officer was assigned as supervisor.

### Study Variables

Dependent variables: Overweight and obesity (BMI Z score for age). Independent variables include: children sociodemographic variables: age and sex; parent’s sociodemographic and economic factors: maternal educational status, marital status, maternal occupational status, father’s occupation, father’s educational status, family size, wealth index, parent’s preference look of their child physical shape, and knowledge of parents on consumption of healthier foods; and behavioral characteristics of children: physical activity level, time spent playing electronic games, and time spent on watching TV/video; and child feeding habit; childs feeding practices and dietary diversity score of children.

### Operational Definition

Preschool children: refers to children aged from 3 to 5 years old [24].
Overweight: defined as preschool children whose BMI for age Z score was between +2 SD and +3 SD [1].
Obesity: defined as preschool children whose BMI for age Z score was above +3 SD [1].
Complementary feeding: the introduction of solid or semisolid foods in addition to breast milk for children.
Dietary diversity score (DDS): it was analyzed and categorized as undiversified if < 4 food categories were consumed per every meal and diversified if ≥ 4 food categories were consumed per every meal [22].
Parental knowledge about the consumption of healthier food: a parent who knows about a variety of foods, including fruits and vegetables, and about the importance of breastfeeding.
Good knowledge about consumption of healthier food: respondents who scored points at the mean and above for the knowledge questions prepared.
Poor knowledge about consumption of healthier food: respondents who scored points below the mean for the knowledge questions prepared.
Snack: A small portion of food or drink or light meal that is eaten between regular meals.

### Data Quality Assurance

Two days training was given for data collectors and supervisors on the data collection methods, mobile data collection platform, ODK, and anthropometric measurement. Standardization was done on 10 children, and data collectors measured those children twice and recorded their results to compare with the principal investigator’s measurement record as a reference. The technical error of the measurement was calculated using Ena Smart software. If any data collector failed to pass the standardization procedure, additional training was given accordingly. Calibration of the weight scale was done by using 1 kg of highland water daily and, after every measurement, putting it to zero to maintain accuracy. Data collectors were daily supervised by the supervisors. All activities were checked by the supervisor and reported to the principal investigator daily. The questionnaire was prepared first in English and then translated into Amharic. Again, it was translated back into English to maintain consistency. Prior to the actual data collection, pre-testing of the questionnaire was carried out on 5% of the sample population. All necessary improvements were made based on the pretest results to avoid any confusion and for better completion of the questions.

### Data Processing and Analysis

The collected data were downloaded from ODK aggregate as an Excel file and exported to SPSS version 25 for analysis. The normality of the data was checked by using the Shapiro-Wilks test. Outliers were checked by putting the data on a whisker box plot. WHO Anthro 2007 version 3.2.2 was used to generate the z-score values for BMI-for-age (BAZ) of preschool children. Then the values were transformed into SPSS for further analysis. Kappa statistics were used to compare parental perceptions about their child’s weight status. Dietary diversity scores were determined by asking the parents to list the food items eaten in the previous 24 h, and the listed food items were categorized into seven food groups based on the WHO Food Group of 2010. The score was categorized as diversified if the child consumes >4 food groups and undiversified if the child consumes <4 food groups [22]. Descriptive statistics like frequency, mean, median; standard deviation, interquartile range, and percentage were used to describe the study population with relevant variables. Binary logistic regression models were used to identify factors associated with overweight/obesity among preschool children. Bivariate analysis was used to differentiate candidate variables for multivariate analysis. All variables with p < 0.25 were entered into the final model. Statistical significance was defined at a probability level of less than 0.05. The AOR along with a 95% CI were used to assess the strength of the association. The Hosmer-Lemeshow goodness-of-fit statistic was used to check model fitness for all models. Multicollinearity was checked by using the variance inflation factor (VIF <10) and tolerance. The maximum VIF value was 5.03, showing no threat of multicollinearity. The household wealth index was constructed by using principal component analysis (PCA) after checking assumptions based on household assets for urban areas, which are taken from the Ethiopian Demographic and Health Survey (EDHS) [25]. The index was categorized into tertiles as low, medium, and high, and households were assigned to each of the categories. Finally, the results of the study were presented using text, tables, and figures.

## Results

### Sociodemographic and Economic Characteristics of Preschool Children

A total of 577 preschool children with their mothers/caregivers (with a response rate of 100%) participated in the study. Of the participants, 53.6% were males and 46.4% were females. The median age of the participants was 45 months (IQR = 39, 53). The majority of mothers/caregivers were married (96.9%), around 32.1% of mothers/caregivers had completed secondary school, and about 43.8% of fathers had completed secondary school. Around 47% of the study participants had less than five household members, and 53.2% had five or more household members. About 34.8% of the respondents were from the medium socioeconomic class, whereas 32.9% were from the high socioeconomic class and 32.2% were from low socioeconomic class (Table 1).

**TABLE 1:** Sociodemographic and economic characteristics of preschool children and their mothers/caregivers in Arba Minch city in 2022 (n = 577). (Predictors of Overweight/Obesity among Preschool Children in Arba Minch City, Southern Ethiopia, 2022: Does Consumption of Sweet Food Predispose to Overweight/Obesity?).

Variables	Attributes	Frequency	Percent (%)
Sex of child	Male	309	53.6
	Female	268	46.4
Age of child (in months)	36–47	306	53.0
	48–60	271	47.0
Educational status of the child	Enrolled to KG	248	43.0
	Not enrolled in KG	329	57.0
Marital status of mothers	Married	559	96.9
	Others^a^	18	3.1
Educational status of mothers	Unable to write and read	103	17.9
	Primary education	163	28.2
	Secondary education	185	32.1
	Above secondary school	126	21.8
Educational status of fathers	Unable to write and read	37	6.4
	Primary education	94	16.3
	Secondary education	193	33.4
	Above secondary school	253	43.8
Occupational status of mothers	Government employee	128	22.2
	Private employee	38	6.6
	Merchant	144	25.0
	Housewife	230	39.9
	Others^b^	37	6.4
Occupational status of fathers	Government employee	209	36.2
	Private employee	70	12.1
	Merchant	228	39.5
	Others^b^	70	12.1
Family size (in number)	<5	307	53.2
	≥5	207	46.8
Wealth index	Low	186	32.2
	Medium	201	34.8
	High	190	32.9

^a^ Single, widowed, divorced.

^b^ daily laborer, gospel preacher; KG, kindergarten.

### Feeding Practices and Dietary Pattern of Preschool Children

**FIGURE 2:**
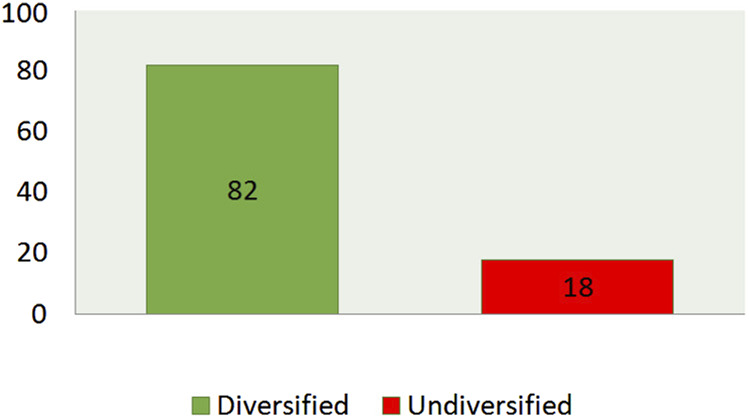
Childs dietary diversity score in the last 24 h preceding the date of survey in Arba Minch city, South Ethiopia, 2022. (Predictors of Overweight/Obesity among Preschool Children in Arba Minch City, Southern Ethiopia, 2022: Does Consumption of Sweet Food Predispose to Overweight/Obesity?).

The majority of children (94.5%) were breastfed, but only 0.5% started breastfeeding immediately after birth. Half of the study participants (50.4%) were given infant formula feeding, and around 41.1% of them started formula feeding between 4 and 6 months of age. Regarding complementary feeding, 48.4% of the participants started complementary feeding at 6 months of age, and 47.5% started at 7 months and above, and 4.2% started before 5 months (Table 2). Dietary diversity scores of preschool children were studied by asking mothers/caregivers about foods taken in the last 24 h. The mean dietary diversity score was 4.73, with a standard deviation of +1.317. About 82% of children consumed four or more food groups (Figure 2). A large number of preschool children consumed other fruit and vegetable groups (97.1%), followed by foods made from grains, roots, and tubers (89.6%), and milk and milk products (88%). Half of the participants consumed sweet foods. 51.8% consumed vitamin A-rich fruits and vegetables, and 51.3% consumed eggs. Meat, poultry, organ meats, and fish were the least consumed food groups (23.2%). The majority of children (77.6%) were fed three or more times daily, but 4.5% were fed two or fewer times daily. Nearly 96% of children preferred to eat snacks. Of this, about 33.3% of children eat a snack once a day (Table 2).

**TABLE 2:** Feeding practice of preschool children in Arba Minch city south Ethiopia, 2022. (Predictors of Overweight/Obesity among Preschool Children in Arba Minch City, Southern Ethiopia, 2022: Does Consumption of Sweet Food Predispose to Overweight/Obesity?).

Variables	Attributes	Frequency	Percentage
Breast feeding	Yes	545	94.5
	No	32	5.5
Time of start of breast feeding after delivery	Immediately with in an hour	3	0.5
	After 1 hour Hours	475	82.3
	A days or after	67	11.6
Infant formula feeding	Yes	291	50.4
	No	286	49.6
Age started infant formula	≤3 months	10	3.4
	4–6 months	237	81.5
	>6 months	44	15.1
Age started complementary feeding	≤5 months	24	4.2
	At 6 months	279	48.4
	≥7 months	274	47.5
Duration of continued breast feeding	<12 months	7	1.2
	12–18 months	368	63.8
	19–24 months	170	29.5
Number of meal per day	Once	15	2.6
	Twice	11	1.9
	Thrice	103	17.9
	More than thrice	448	77.6
Eat snacks	Yes	554	96.0
	No	23	4.0
Number of snacks per day	Once	192	34.7
	Two times	24	4.3
	Three times	40	7.2
	More than three times	298	53.8

### Physical Activity and Sedentary Behavior of Preschool Children

Nearly half (45.4%) of the study participants watched TV/video. Of those, 6.4% spent two and more than 2 hours a day, and 1.2% spent two and more than 2 hours playing mobile and other electronic games (Table 3).

**TABLE 3:** Physical activity and sedentary behavior of preschool children, parental preference about their children’s weight, and knowledge on consumption of healthier food in Arba Minch city in 2022 (Predictors of Overweight/Obesity among Preschool Children in Arba Minch City, Southern Ethiopia, 2022: Does Consumption of Sweet Food Predispose to Overweight/Obesity?).

Variables	Attributes	Frequency	Percentage (%)
Moderate sport activities for at least 30 min per day	Not at all	101	17.5
	1-2 times	88	15.3
	3–5 times	179	31.0
	All the week	209	36.2
Time spent on watching TV/video per day	<2 h	225	39.0
	≥2 h	37	6.4
Time spent playing mobile/ computer games per day	<2 h	106	18.4
	≥2 h	7	1.2
Physical education class	Given	227	39.3
	Not given	21	3.6
Travel by walking to and from school per day	<30 min	127	22.0
	≥30 min	45	7.8
Travel by cycling to and from school per day	<30 min	52	9.0
	≥30 min	24	4.2
Preference about their children weight	Underweight	140	24.3%
	Normal	433	75%
	Overweight	4	0.7%
Knowledge on consumption of healthier food	Poor	435	75.4
	Good	142	24.6

### Parental Preference About Their Children’s Weight and Knowledge on Consumption of Healthier Food

Only 0.7% of overweight children were correctly described as overweight by their parents, whereas 69.6% of parents described their children under the normal weight category, and 6.6% were described as underweight by their parents. Kappa statistics were used to compare the parental perception and the actual measured BMI of the children. It shows no agreement between parental perception and the actual measured BMI of children. (k-value −0.14, p < 0.260). The mean knowledge score of mothers and caregivers on the consumption of healthier food was 3.56. The majority of parents (75.4%) scored below the mean value. In terms of feeding healthier foods to their children, 24.6% of parents were knowledgeable (Table 3).

### Prevalence of Overweight/Obesity Among Preschool Children

The combined prevalence of overweight/obesity was 23.9% (95% CI: 20.5, 27.6). Of this, 15.6% (95% CI: 12.7, 18.8) were overweight and 8.3% (95% CI: 6.2, 10.9) were obese. Moreover, in the current study, the prevalence of underweight, stunting, and wasting was 6.6% (95% CI: 4.7, 8.9), 35.9% (95% CI: 32, 39.9), and 6.2% (95% CI: 4.4, 8.5), respectively.

### Factors Associated With Overweight/Obesity Among Preschool Children

In this study, factors that were related to overweight/obesity (P-value <0.25) in the bivariate analysis were consumption of sweet foods, time spent watching TV, age of the child in months, the mother’s educational status, the father’s educational status, family size, and snack preference. According to the results of multivariable logistic regression, factors that have a significant association with the outcome variable are the age of the child in months, family size, consumption of sweet foods, and time spent watching TV/video.

Preschool children with the age of 36–47 months had a 2.19 (AOR = 2.19; 95% CI: 1.10, 4.35) times higher risk of being overweight or obese compared to children with the age of 48–60 months. Moreover, the result shows that preschool children with family size less than five were 2.33 (AOR = 2.33; 95% CI: 1.13, 4.82) times more likely to be overweight/obese as compared with children with family sizes of five and above. Children who spent 2 h or more watching TV/video in a day had a 2.79 (AOR = 2.79; 95% CI: 1.13, 6.90) time increased risk of becoming overweight/obese than participants who spent less than 2 h. The odds of being overweight/obese were 4.14 (AOR = 4.14; 95% CI: 1.86, 9.20) times higher for those children who consume sweet foods compared with those who don’t consume sweet foods (Table 4).

**TABLE 4:** Factors associated with overweight/obesity among preschool children in Arba Minch City in 2022 (n = 577) (Predictors of Overweight/Obesity among Preschool Children in Arba Minch City, Southern Ethiopia, 2022: Does Consumption of Sweet Food Predispose to Overweight/Obesity?).

Variables	Attributes	Overweight/obesity		COR (95% CI)	AOR (95% CI)
		Yes	No		
Age of the child (in months)	36–47	84	222	1.52 (1.03, 2.25)	2.19 (1.10, 4.35)**
	48–60	54	217	1	1
Family size (in number)	<5	66	241	1.32 (0.91, 1.95)	2.33 (1.13, 4.82) **
	≥5	72	198	1	1
Time spent on watching TV/ video	<2hrs	48	177	1	1
	≥2hrs	22	15	5.41 (2.61, 11.22)	2.79 (1.13, 6.90)**
Snack preference	Yes	128	427	0.36 (0.15, 0.85)	0.19 (0.03, 1.16)
	No	10	12	1	1
Mother educational status	Unable to write and read	38	65	1.52 (0.87, 2.66)	1.88 (0.61, 5.75)
	Primary education	32	131	0.63 (0.37, 1.10)	1.05 (0.32, 3.48)
	Secondary education	33	152	0.86 (0.32, 0.97)	1.39 (0.49, 3.96)
	Above secondary school	35	91	1	1
Fathers educational status	Unable to write and read	15	27	1.5 (0.76, 3.01)	1.00 (0.26, 3.93)
	Primary education	12	82	0.40 (0.20, 0.78)	0.16 (0.04, 0.71)
	Secondary education	43	145	0.81 (0.52, 1.25)	0.75 (0.31, 1.83)
	Above secondary school	68	185	1	1
Consumption of sweet food	Yes	94	208	2.37 (1.58, 3.55)	4.14 (1.86, 9.20)*
	No	44	231	1	1

*P-value <0.05; **P-value <0.01; CI, confidence interval; AOR, adjusted odds ratio; COR, crude odds ratio.

## Discussion

This study was aimed at assessing the prevalence of overweight/obesity among preschool children in Arba Minch city. According to the study, the combined prevalence of overweight/obesity among preschool children was 23.9% (Overweight: 15.6% and obesity: 8.3%).

The result of the current study was comparable with a study done in Nepal, which was 25.9%, Vietnam 21.1%, and Brazil 24.4% [16, 17, 26]. This shows that the burden of the problem increases in both developing and developed countries in an equal manner. It might be due to lifestyle changes, increased use of motor-based transportation, and a sedentary lifestyle.

Although the finding of this study was higher than the evidence from studies conducted in Asian countries, 5.8% [27], central parts of Iran, 15.5% [28], sub-Saharan Africa, 6.8% [15], Kenya, 13.4% [29], and Ghana, 1.5% [18]. Moreover, the prevalence of overweight/obesity reported in the current study was higher than the prevalence reported earlier in Dire Dawa, which was 16.6% [7] and 13.8% in Gondar City [6]. This discrepancy might be due to the effect of urbanization on people’s nutritional styles. In addition, the difference might be due to difference in study participants as study in Kenya included children age 3–6 years [29], that of Iran included adolescents [15], and Asia included 5–11 years [27]. Moreover, it might be because of dissimilarity in sociodemographic characteristics of respondents, change in people’s lifestyles, and difference in study settings as study done Iran [15] was done at clinics. Furthermore, the discrepancy could be also due to difference in study design as previous studies were systematic [27] and scoping reviews [15]. The prevalence of overweight/obesity in this study was lower than that of a study conducted in South Carolina, which was 34.4% [30]. This disparity might be due to the variation in study design that the studies used.

The study indicated that the highest prevalence of overweight/obesity was observed among children within the 36–47 months of age group (27.5%), of whom 18.3% were overweight and 9.2% were obese, and the smallest prevalence was among the 48–60 age group (19.9%). Similar results were seen in a study done in Gondar City [6]. This may be due to having less time to play outdoors and spending more time at home than in the 48–60 age group. Moreover, they had a lower chance of getting to school at this age.

Children from families that had fewer <5 family members were two times more likely to be overweight, overweight/obese than from families that had five or more family members. These findings are consistent with those from a study done in Bahir Dar [5]. This might be related to a reduced tendency to share the available resources at home, the family spending more time and attention to care for their child, and the family having a good possibility of getting good nutrition.

Time spent watching TV or video for more than 2 hours was one of the factors associated with overweight/obesity in this study. By this, the same results were observed in studies conducted in Gondar [6], Poland [31], the central part of Iran [28], and Duwakot, Bhaktapu [17]. This might be because too much screen time can limit the physical activity level of the children and increase the chance of getting snacks which may have high calories with low nutrient density. At the same time, it may take their sleep time and aggravate hormonal imbalance causing slow metabolic rate which can lead to weight gain and obesity [32].

In the present study, overweight/obesity was also associated with the consumption of sweet foods. Children who consumed sweet foods had four times greater odds of being overweight or obese than children who did not consume them. The same result was shown in studies conducted in Ethiopia [6, 8]. It might be due to the growing habit of using sugar and sweet drinks as part of food, which have high calories but low nutrient density, which disturbs sugar regulation and results in the release of a high amount of insulin, which activates the storage of glucose, putting the children at risk of getting overweight [33].

### Program and Policy Implications

The current study finding indicated a high prevalence of overweight/obesity among preschool children was predicted by different factors. This findings are crucial for program, policy, and practice in the city, aligning with the Sustainable Development Goal (SDG) 2 particularly target .2.2 aiming at ending malnutrition including overweight among children by 2023 [34]. Therefore, programmers and policymakers should focus on reduction of overweight/obesity to achieve SDG 2 via comprehensive implementation of appropriate interventions targeted on identified factors. In addition, this significant public health problem needs a consistent and integrated intervention from city, zonal, and regional health bureau as well as other stakeholders by giving due emphasis to early detection of malnutrition and designing control and prevention measures to reduce overweight/obesity among preschool children.

### Strengths and Limitations

The current study used a large sample size which supports the findings precision. A practical training was given on anthropometric measurements, instrument calibration, and pretesting data collection tool. In addition, standard data collection tool were used for dietary diversity and physical activity measurement. However, it is difficult to establish temporal relationship due to the cross-sectional nature of the study. Moreover, even if the data were collected by trained people, there might be a risk of recall bias and social desirability bias by mothers/caregivers on dietary data and the dietary data did not consider portion size.

### Conclusions

The effect of double burden of malnutrition is evident in the study area among preschool children with a high prevalence of overweight/obesity. Being in the age group of 36–47 months, having a family size of less than five, spending more than 2 h watching TV or video, and consuming sweet foods were found to be the factors associated with overweight/obesity among preschool children in this study. Special attention should be given to child feeding practices and reducing sedentary lifestyles. Besides, these, proper diagnostic criteria and treatment plans should be applied, and follow-up should be increased to reduce the prevalence. Researchers should conduct a longitudinal prospective cohort study to address further clinical and socio-demographic characteristics of children in relation to overweight/obesity.

## Abbreviations

AOD, Adjusted odds ratio; BAZ, Body mass index for age; BMI, Body mass index; CI, Confidence interval; COR, Crude odds ratio; DDS, Dietary diversity score; DHS, Demographic health survey; EDHS, Ethiopian demographic health survey; IQR, Inter quartile range; KG, Kinder garden; ODK, Open data kit; PCA, Principal component analysis; SD, Standard deviation; WHO, World health organization.
